# Effects of microplastics on *Daphnia*-associated microbiomes in situ and in vitro

**DOI:** 10.1093/ismejo/wrae234

**Published:** 2024-12-12

**Authors:** Anna Krzynowek, Broos Van de Moortel, Nikola Pichler, Isabel Vanoverberghe, Johanna Lapere, Liliana M Jenisch, Daphné Deloof, Wim Thielemans, Koenraad Muylaert, Michiel Dusselier, Dirk Springael, Karoline Faust, Ellen Decaestecker

**Affiliations:** Department of Microbiology, Immunology and Transplantation, Laboratory of Molecular Bacteriology (Rega Institute), KU Leuven, Herestraat 49, Leuven 3000, Belgium; Department of Biology, Laboratory of Aquatic Biology, MicrobiomeEcoEvo group, KU Leuven, Etienne Sabbelaan 53, Kortrijk 8500, Belgium; Department of Biology, Laboratory of Aquatic Biology, MicrobiomeEcoEvo group, KU Leuven, Etienne Sabbelaan 53, Kortrijk 8500, Belgium; Department of Biology, Laboratory of Aquatic Biology, MicrobiomeEcoEvo group, KU Leuven, Etienne Sabbelaan 53, Kortrijk 8500, Belgium; Department of Biology, Laboratory of Aquatic Biology, MicrobiomeEcoEvo group, KU Leuven, Etienne Sabbelaan 53, Kortrijk 8500, Belgium; Department of Microbial and Molecular Systems (M^2^S), Center for Sustainable Catalysis and Engineering (CSCE), KU Leuven, Celestijnenlaan 200f, Leuven 3001, Belgium; Instituut voor Landbouw-, Visserij- en Voedingsonderzoek / Flanders Research Institute for Agricultural, Fisheries and Food, Jacobsenstraat 1, Oostend 8400, Belgium; Department of Chemical Engineering, Sustainable Materials Lab, KU Leuven, Etienne Sabbelaan 53, Kortrijk 8500, Belgium; Department of Biology, Laboratory of Aquatic Biology, MicrobiomeEcoEvo group, KU Leuven, Etienne Sabbelaan 53, Kortrijk 8500, Belgium; Department of Microbial and Molecular Systems (M^2^S), Center for Sustainable Catalysis and Engineering (CSCE), KU Leuven, Celestijnenlaan 200f, Leuven 3001, Belgium; Department of Earth and Environmental Sciences, Soil and Water Management, KU Leuven, Kasteelpark Arenberg 20, Leuven 3001, Belgium; Department of Microbiology, Immunology and Transplantation, Laboratory of Molecular Bacteriology (Rega Institute), KU Leuven, Herestraat 49, Leuven 3000, Belgium; Department of Biology, Laboratory of Aquatic Biology, MicrobiomeEcoEvo group, KU Leuven, Etienne Sabbelaan 53, Kortrijk 8500, Belgium

**Keywords:** Microplastics pollution, freshwater ecology, *Daphnia magna*, host microbiome

## Abstract

Microplastic pollution in aquatic environments is a growing global concern. Microplastics, defined as plastic fragments smaller than 5 mm, accumulate in freshwater reservoirs, especially in urban areas, impacting resident biota. This study examined the effects of microplastics (MP) on the performance and microbiome of *Daphnia*, a keystone organism in freshwater ecosystems, through both in situ sampling of freshwater ponds and a controlled 23-day in vitro exposure experiment. Using bacterial 16S ribosomal RNA gene amplicon sequencing and whole-genome shotgun sequencing, we analyzed the microbiome's composition and functional capacity in relation to microplastic pollution levels. Urban ponds contained higher microplastic concentrations in water and sediment than natural ponds, with distinct differences in plastic composition. Bacterioplankton communities, defined as bacterial assemblages in the water column, were more diverse and richer than *Daphnia*-associated microbiomes. Overall, the in situ study showed that the composition of the *Daphnia-*associated community was influenced by many factors including microplastic levels but also temperature and redox potential. Functional analysis showed increased relative abundances of polyethylene terephthalate degradation enzymes and antibiotic resistance genes in microbiomes from high-microplastic ponds. In the in vitro experiment, the bacterioplankton inoculum source significantly influenced *Daphnia* survival and microbiome composition. Network analysis identified specific taxa associated with MP within the *Daphnia* microbiome. Our findings highlight that urbanization leads to higher microplastic and antibiotic resistance gene burdens, influencing host-associated microbiomes through taxonomic shifts, functional enrichment, and survival outcomes, with potential implications for the resilience of aquatic ecosystems.

## Introduction

By 2030, the world’s aquatic environment is predicted to accommodate more than 80 million metric tons of plastic waste [1, 2]. Microplastics (MP), i.e., plastic particles smaller than 5 mm originating from either fragmentation of larger plastic materials or as such released in the environment, have become of major environmental concern not only in marine but also in freshwater ecosystems [3]. In particular, stagnant ponds near densely populated areas are prone to high plastic deposition burdens showing concentrations of up to 19 860 particles/m^3^ [3–5]. Although studies have mainly focused on the impact of MP on marine organisms, MP also affect freshwater organisms at different levels of the trophic chain [6]. This includes members of the zooplankton genus *Daphnia,* which occupy an important lower trophic level within aquatic food webs. *Daphnia* feeds on small suspended particles in the water and sediments in freshwater ecosystems resulting into MP accumulation upon ingestion [7–9]. Acute effects of MP exposure have been observed in *Daphnia,* and include molecular responses [10–12] effects on survival [11, 13, 14], reproduction [11, 14–17], feeding rates [13, 18], body mass [14], and mobility [8, 19].

*Daphnia*’s carapax, body cavities, gut, and feeding apparatus are colonized by bacteria, collectively defined as the microbiome [20–22]. The assembly of this microbiome involves complex interactions with the surrounding bacterioplankton and is influenced by the host’s diet [23, 24], genetic background [24–26], and environmental conditions [27, 28]. The host's microbiome contributes to the host's fitness [29]. Moreover, key members of *Daphnia*'s microbiome (defined as taxa consistently present across different individuals and environments) have been identified [22], including certain taxa that play critical roles in host physiology, with positive correlations to increased body size, growth, and survival [20, 29]. Additionally, the gut microbiome is suggested to play a role in the degradation of toxins, such as those produced by cyanobacteria [26, 30]. Thus, *Daphnia*’s microbiome not only contributes to host survival but may enhance the host's ability to tolerate and restrain environmental stressors.

Studies that address the impact of MP pollution on the *Daphnia* microbiome are limited. A recent short-term study on polystyrene (PS) particles showed a transfer of microbiota from *Daphnia magna* to MP biofilms [31], which are essential hubs for plastic polymer biodegradation [32, 33]. *Vice versa*, the biofilm community may also interact with the already established microbiome community. In this scenario, specific members of the so-called plastisphere (i.e. microbial community living on microplastic surfaces) may be transferred to the microbial community of particle-feeding zooplankton upon ingestion of the plastic particles. With the transfer of these members comes the transfer of some genes associated with MP. These include plastic polymer degradation enzymes and antibiotic resistance genes (ARGs), which have been demonstrated to spread via MP-associated biofilms [34, 35].

**Figure 1:**
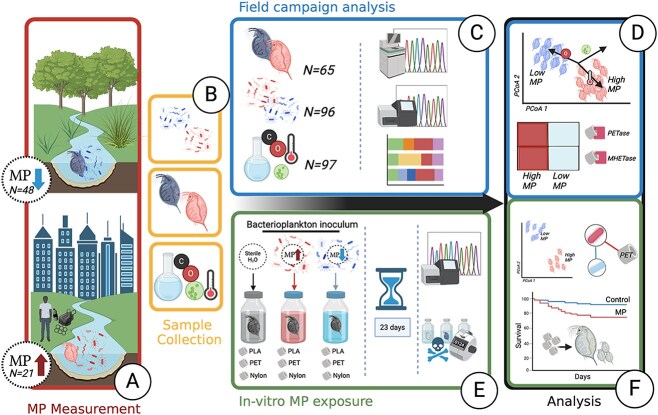
**Graphical overview of the study design and experiments. A.** Samples of sediment cores and water were collected from two types of ponds, natural lakes (N = 24,24 for water, sediment) and urban ponds (N = 12,9 for water, sediment), for MP quantification. Additionally, the following data were collected: **B.** Pond bacterioplankton, local *Daphniidae* populations, and environmental factors. **C.** Eight water parameters (N = 97) were compared across sampled ponds; bacterioplankton (N = 96) and *Daphniidae* microbiome samples (N = 65), were sequenced with 16S rRNA for microbial community analysis and whole genome shotgun sequencing at high depth (1 million reads per sample) for functional analysis. **D.** The community composition of bacterioplankton and whole-*Daphnia* microbiomes was compared between high and low MP ponds, while accounting for sampled environmental factors. Furthermore, the functional capacity of host-associated microbiomes, including the average abundance of PET-degrading enzymes and antibiotic resistance genes (ARGs) was compared between the two environments. **E.** In the short-term exposure experiment, three *Daphnia magna* genotype clones were exposed to PET, PLA, and nylon microplastic fibres for 23 days. The exposure jars were inoculated with bacterioplankton from either the high-MP BP pond or the low-MP DG pond, collected during the field campaign. After 23 days, the DNA from both animals and bacterioplankton was extracted and sequenced (16S rRNA gene amplicon sequencing). Data on population and animal survival were collected throughout the experiment. **F.** The community composition of bacterioplankton and whole-*Daphnia* microbiomes was compared between different MP exposures (PET, PLA, nylon) and non-exposed animals (negative control). Additionally, an association network between taxa and MP was constructed to identify taxa significantly associated with specific MP. Finally, *Daphnia* survival in response to MP exposure was analysed throughout the experiment. (Created in BioRender. Faust, K. (2024) https://BioRender.com/j85l656)

Here, we studied the effects of MP on *Daphnia’s* microbiome in situ by sampling freshwater reservoirs and in vitro by conducting a 23-day MP-exposure experiment (Fig. 1). In the in situ study, we measured the prevalence of MP across urban and natural freshwater ponds in Flanders, Belgium using 16S rRNA gene amplicon and whole genome shotgun sequencing. We investigated the microbial community composition and functional capacity of the bacterioplankton and native *Daphnia* populations, which were then correlated with MP pollution levels. The field campaign results were compared to those of the in vitro exposure experiment in which we cultured different *D. magna* clones with PET, PLA (polyester), and Nylon (polyamide) MP for 23 days and monitored *Daphnia* population parameters. The microbiome community composition was resolved using 16S rRNA gene amplicon sequencing and associations of taxa with MP exposure were investigated.

## Materials and methods

### Field sampling campaign

#### Pond sampling

Bacterioplankton, *Daphnia* population and MP were sampled from twelve ponds located in Flanders, Belgium (Supplementary Fig. 1)*.* The ponds were categorized as natural lakes or city ponds based on their location (Supplementary Table 2). On site measurements of temperature, pH, oxygen, redox, and conductivity were carried out in triplicate using a Hach HQ40d multi-meter. NPOC concentrations were measured in the lab with a Shimadzu TOC-L analyzer (Supplementary Table 3). A total of 161 samples of *Daphnia* (65) and bacterioplankton (96) were collected. To recover *Daphnia*, each 30 L of sampled water was filtered through a 300 μm zooplankton net, rinsed, dried, and frozen at −80°C. For bacterioplankton, 250 ml water samples were collected at three depths (bottom, middle, top) and filtered through 0.22 μm sterile filters, were subsequently frozen at −80°C until processing. MP were sampled from both sediment and water columns in triplicate and within 1-metre distance from each other. To collect microplastics (sized 64 μm–250 μm) in the pond water column, 150 L sample was passed through stacked metal sieves of decreasing mesh sizes into a clean glass jar. To collect from the pond sediment, we sampled the top 5 cm of solid sediment (or ~500 ml of loose sediment when solid sediment was not available) and isolated microplastics as described below. All sediment and water samples were handled exclusively with non-plastic equipment.

#### MP quantification and characterization

MP particles from the water in the glass jars and from sediment samples were recovered according to a previously published method [36, 37]. The process involved three steps: digestion of organic matter with H_2_O_2_, staining with Nile Red-dye, and applying a machine-learning algorithm to distinguish and count plastic particles (Supplementary materials and methods).

#### Molecular biology

DNA from bacterioplankton and whole *Daphnia* samples were extracted with NucleoSpin (Macherey-Nagel) kit following the manufacturers' protocols. Extracted DNA was resuspended in a 20 μl elution buffer, quantified with a NanoPhotometer N60 and used for 16S rRNA gene amplicon and whole-genome shotgun sequencing. The V4 region of the 16S rRNA gene was amplified using a nested PCR strategy as detailed in Supplementary materials and methods and sequenced using the MiSeq platform (Illumina). Shotgun libraries were prepared with the KAPA HyperPrep PCR-free Kit (Roche) and sequenced on the NovaSeq 6000 (Illumina) platform at 100 Gb per sample at the KULeuven Genomics Core, Belgium.

#### Analysis of MP distribution in nature

Average MP concentrations between urban and natural pond categories were compared in sediment and water samples. All calculations were performed in R-studio version 4.3.1 or python version 3.11.7. Box plots were generated using ggplot2 and ggpubr package. The data were assessed for normality using the Shapiro–Wilk test followed by a Wilcoxon rank sum test to check for statistically significant differences between average MP in urban and natural ponds. The differences in composition were tested with Multivariate Analysis of Variance (MANOVA) using Python's statsmodels library providing four test statistics: Wilks' lambda, Pillai's trace, Hotelling-Lawley trace, and Roy's greatest root, to assess the multivariate effect of pond category on the composition of MP concentrations.

#### Analysis of 16S rRNA amplicon sequencing for quantifying differences in community composition of *daphnia* and bacterioplankton samples

The quality of demultiplexed paired-end reads was assessed using FastQC [38]. The run generated 10 832 180 raw sequences (min 7781, max 55 923 per sample). Paired-end sequences were filtered, denoised, and dereplicated with DADA2 [39] in R-studio. Taxonomy was assigned using the Silva 138.1 SSU Ref NR 99 database [40]. Reads labelled as “mitochondria” or “chloroplast” were removed. The ASV table, taxonomy, and metadata were imported into a phyloseq object (v. 1.44.0, [41]). A rarefaction curve was constructed using the vegan package (v. 2.6–4, [42]), and samples were rarefied to the depth of the sample with the fewest reads (4664), removing 2007 ASVs out of the total of 8237 ASVs. Bray-Curtis dissimilarities were computed for ordination. For distance-based redundancy analysis (db-RDA), highly collinear variables (*r* > 0.7) were removed and remaining ones standardized using z-score standardisation. The significance of the association between environmental variables and community composition was tested with permutation tests (alpha = 0.05). Diversity indices were calculated using alpha and boxplot_alpha functions from the microbiome package (v. 1.22.0, [43]).

#### Analysis of whole-genome shotgun of whole *Daphnia*-microbiome samples

Demultiplexed paired-end reads were quality-checked using FastQC, adapter sequences were trimmed, and reads with a Phred score below 30 were filtered out using Fastp [44], resulting in 12.59 billion high-quality reads (14 330 348 to 261 293 254 per sample). Eukaryotic sequences were identified and removed with Kraken2 v.2.1.3 [45] using the NCBI non-redundant nucleotide database. Reads mapping to genomes of Cladocerans, *Daphnia*, microsporidia, and *Homo sapiens* were removed from *Daphnia* microbiome samples, while reads mapping to freshwater algae were removed from bacterioplankton samples. Bowtie2 [46] and Samtools [47] were used for this filtering process. Bacterial reads were co-assembled using MEGAHIT [48]. Contigs were checked against the Refseq bacteria database with Kraken2 and only bacterial contigs were analysed. Open Reading Frames (ORFs) were predicted with Prodigal [49]. Quality-filtered reads were aligned to contigs using Bowtie2 and alignment statistics were obtained with Samtools. FeatureCounts [50] extracted read counts for each ORF. Gene abundances were normalized using the adapted RPKM formula [51]:


$$ \frac{Mapped\ Reads}{\frac{Totel\ Reads}{\mathrm{1,00,000}}\ast ORF\ Lenght} $$

Proteins were annotated with eggNOG-mapper v2.1.6 [52], and abundances were derived using FeatureCounts. Differences in gene distributions were visualized using a constrained ordination on the Bray–Curtis dissimilarity matrix with counts data normalized to proportions. Functional enrichment between high and low MP groups was assessed using DESeq2 [53]. PET degradation pathways were compared by mapping KO identifiers against the KEGG database [54]. ARGs were identified by aligning protein sequences against the CARD database [55] using BLASTP [56] with stringent criteria (an e-value threshold of 1e-5, a minimum query coverage of 70%).

### In vitro *D. magna* exposures to MP

#### MP fibre generation

Three types of polymers were used, i.e, the poorly biodegradable polyethylene terephthalate (PET), polyamide 6,6 (Nylon), and (under industrial composting conditions) biodegradable alternative polylactic acid (PLA) [57]. All three polymers are used in textile manufacturing and their MP are found in waste waters from textile fabrics and household washing activities. PET and Nylon fibres (diameter 0.01 mm) were procured from Goodfellow (GF01552395 and GF15603331, respectively) and PLA filaments (0.05 mm diameter) were kindly provided by Sioen Industries (Ardooie, Belgium). Uniformly sized microfiber preparations were obtained as described by Cole et al. (2016), including solidifying them at −80°C, cutting them into 50 μm pieces, and processing them with heating and vacuum filtration (Supplementary materials and methods). The fibres were sterilized with ethanol and stored in a sterile container until use.

#### Determining optimal MP concentrations

To determine an optimal concentration of MP in the exposure experiment, concentrations of 12 mg/L, 50 mg/L, and 100 mg/L of PET, PLA, and Nylon fibres were tested for acute (48 hours) and chronic (23 days) effects on three *D. magna* clones (KNO15.04, F, and OM2.11) (Supplementary materials and methods). A concentration of 2.5 ± 0.1 mg/L was selected for the main experiment, which was significantly above typical field conditions (0.021 mg/L for Flemish surface water) [58].

#### *D. magna* clones

For the exposure experiment, three *D. magna* clonal lineages were used: F [59], KNO15.04 (from a fishless farm pond near Knokke, 51°20′05.62″ N, 03°20′53.63″ E), and BP (collected from the "Blauwe Poort" (BP) pond, 50.8160° N, 3.2722° E). These genotypes were maintained for many generations at the IRF life sciences laboratory in Kortrijk as reported [60]. At the start of the experiment, 10 hatched neonate *Daphnia* individuals less than 24 hours old were transferred with glass pipettes from the petri dishes into the experimental jars.

#### Experimental design

To compare the effects of bacterioplankton inocula from low MP and high MP ponds on the *Daphnia* microbiome community and on *Daphnia* survival we used pond water taken as 18 L samples from De Gavers (DG) lake and from the plastic-polluted city pond BP as bacterioplankton inocula in the exposure experiments. To remove grazers and dirt, the water was passed through two filters of 100 μm and 10 μm mesh size. Temperature (°C), oxygen concentration (mg/L), oxygen saturation (%), pH, conductivity (μs/cm) and redox (mV) were determined in the two water sources before the experiment (Supplementary materials and methods). The experiment was set up in 500 ml glass jars in triplicate for genotype (F, KNO 15.04, BH, No *Daphnia*) × MP (PET, Nylon, PLA, No MP) × pond microbial inoculum (DG, BP, sterilized H_2_O) yielding 99 jars in total (Supplementary materials and methods). The jars were sterilized by autoclaving and washed with ethanol and purified MQ water to remove any existing MP contamination. Afterwards, they were filled with 400 ml of the bacterioplankton inoculum and 10 *Daphnia* neonates and 1 ± 0.1 mg of microfibers were added. All jars were covered with tin foil to prevent water evaporation and contamination with external microfibers.

#### Data collection

The survival of 10 *Daphnia* individuals was checked every two days. Body size was measured on days 7 and 14 for five randomly selected adults using a stereomicroscope (Olympus SZX10) and ImageJ software. The date and body size of the first brood were recorded by measuring five random juveniles. On days 3, 7, and 14, five *Daphnia* were examined for ingested MP fibres using a light microscope (Olympus BX51) (Supplementary materials and methods). The experiment lasted 23 days, with *Daphnia* fed at a feeding regime of 2 × 10^5^ cells/ml of *Chlorella vulgaris* every two days and of 1 × 10^5^ cells/ml towards the end. Jars were shaken every two days. On day 23, five adults and five juveniles from each jar were harvested, dried, and stored at −80°C for microbiome analysis. For bacterioplankton analysis, 50 ml of water was filtered through a sterile 0.22 μm filter and the recovered cells stored at −80°C.

#### Molecular biology

A total of 246 samples were processed for DNA extraction. The samples were split into bacterioplankton (101 samples) and *Daphnia* (juveniles: 41 samples, adults: 102 samples). DNA from bacterioplankton and whole *Daphnia* samples was extracted with Powersoil Pro (Qiagen) and NucleoSpin (Macherey-Nagel) kits, respectively, following the manufacturers' protocols. Protocols for 16S rRNA gene amplicon sequencing were identical to those used for the samples collected from the field.

#### 16S rRNA gene amplicon sequencing of the MP exposure experiment

For 16S rRNA gene amplicon sequencing, demultiplexed paired-end reads were imported and assessed with FastQC, resulting in 6 901 562 raw sequences (min 92, max 23 577 per sample). Sequences were filtered, denoised, and dereplicated with DADA2 [39] in R-studio. Taxonomy was assigned using the Silva 138.1 SSU Ref NR 99 database [40]. Contaminating taxa were removed using the microDecon package (v. 1.0.2, [61]). Two DNA extraction controls were used to detect contamination. Sequences annotated as “mitochondria” or “chloroplast” were removed. The ASV table, taxonomy, and metadata were imported into R with the phyloseq package (v1.44.0, [41]). Rarefaction curves were constructed, and ASV counts were rarefied to 2494 reads, resulting in the loss of 3010 ASVs out of the total of 5510 ASVs. Significant effects were assessed using permutational multivariate ANOVA (permanova) with homogeneity of variances evaluated using the betadisper function from the vegan package [42].

#### Life-history parameters of *D. magna* in response to MP exposure

Survival curves were calculated based on four time points with the Kaplan–Meier estimator, using the survfit() function in the survival R package. Survival curves were stratified by pond type, fibre type, and *D. magna* genotype using ggsurvplot(), with significance tested by a log-rank test (alpha = 0.05). A Cox proportional hazards model (coxph()) assessed multiple factors' impact on survival, including pond type, genotype, fibre type, and replicate. Statistical significance was evaluated with Wald tests, likelihood ratio tests, and score tests. The impact of pond type, genotype, and fibre type on *Daphnia* body size was assessed with generalized linear models (GLMs) implemented in the glm package, using a Gamma distribution and log link for body size and a Poisson distribution for the age at first brood. Models' goodness of fit was assessed by comparing null and residual deviances, Akaike Information Criterion (AIC), and Fisher Scoring iterations.

#### Co-occurrence network analysis

The networks for DG- and BP-inoculated *Daphnia* microbiome communities from the exposure experiment were constructed to identify significant taxon-MP associations (Supplementary Fig. 4). Prior to network inference, the rarefied ASV counts were subjected to a 10% prevalence filter to remove sparse taxa, and categorical metadata variables were one-hot encoded. The networks were constructed using FlashWeave (v0.19.2, [62] in sensitive mode to ensure accurate detection of direct conditional dependencies between taxa. FlashWeave applies the clr transformation to account for compositionality, and in sensitive mode, it uses partial correlation tests with Fisher’s z-transformation to identify statistically significant associations as well as correcting for potential indirect associations caused by shared environmental factors. For increased reliability, associations were computed only when the ASV count exceeded a threshold (automatically determined by the software). All *P* values associated with network edges were corrected for multiple testing using the Benjamini-Hochberg procedure [63], ensuring that only significant associations were retained (*P* value <.05). The resulting networks were exported in GML format and visualized using Cytoscape (v3.10.1). The node sizes were scaled by the relative abundance of the taxon and coloured by origin (pre-exposure versus pre/post-exposure). The edges are coloured by weight, which corresponds to partial correlation coefficients scaled from −1 (negative association) to 1 (positive association).

## Results

### Urban ponds accumulate more MP than natural lakes

**Figure 2:**
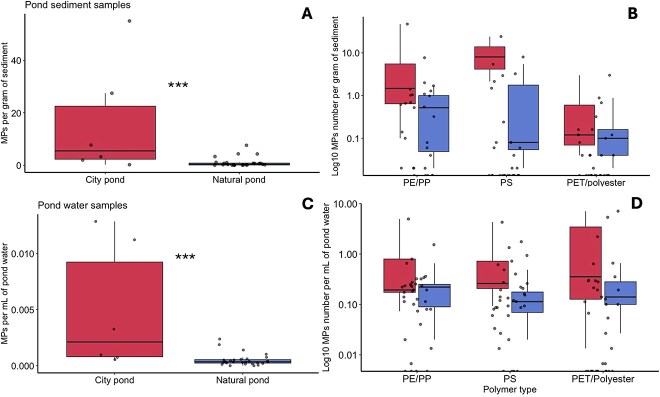
**MP analysis collected from the pond water and sediment. A.** Box plots showing the distribution of MP concentrations measured in the sediment from urban ponds and natural lakes, with the median and interquartile range displayed and samples overlaid. **B.** Box plots showing the concentrations of MP per gram of sediment (n = 3) for each MP type measured in the study. **C.** Box plots showing the distribution of MP concentrations measured in water samples from artificial city ponds (red) and natural lakes (blue), with the median and interquartile range displayed. D. Box plots showing the concentrations of MP per millilitre of water sampled (n = 3) for each MP type measured in the study. *** - indicates statistical significance of a Wilcoxon rank sum test (alpha = 0.05).

Higher MP concentrations were found in city pond sediments compared to natural pond sediments (Two sample T-test, *P* value <.01) (Fig. 2A). At the level of individual ponds, two urban ponds, *Kluizen* and *Blauwe Poort*, showed the highest MP concentrations in the sediment with an average of 21.56 and 10.31 MP per gram of sediment, respectively. *Meer van Rotselaar* and *Reserve Kulak*, two natural lakes, contained the lowest concentrations with an average of 0.08 and 0.16 MP per gram of sediment, respectively (Supplementary Table 1). Similarly, in the water samples, higher MP concentrations were found in the urban ponds than in the natural lakes (Wilcoxon rank sum test, *P* value <.01) (Fig. 2C) with the highest average MP concentration measured in an urban pond (*Citadelpark,* 0.00911 MP in ml of sample) and the lowest in a natural lake (*Meer van Rotselaa*r, 0.0001467 MP per ml of water) (Supplementary Table 1). The composition of MP varied across the samples per pond (water versus sediment) and pond type (urban versus natural). Polyethylene/Polypropylene (PE/PP) were the most abundant MP types in the urban pond sediments followed by Polystyrene (PS), and PET/Polyester (Fig. 2B). In contrast, PET/Polyester was more abundant in water samples (2B,D). Based on the results of the MP analysis, we grouped the ponds into two categories: high and low MP levels for further analysis.

### Host-associated microbiome composition in *Daphnia* linked to environmental variables

**Figure 3:**
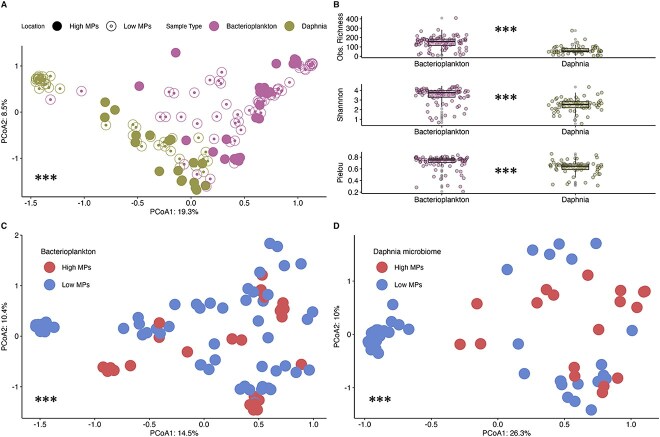
**16S rRNA marker gene analysis of host-associated microbiome and bacterioplankton communities. A.** PCoA ordination showing Bray-Curtis dissimilarities between bacterioplankton and host-associated microbiome samples and shapes depicting the type of the pond (high vs low MP). **B.** Comparison of diversity indices between bacterioplankton and *Daphnia* microbiome samples. Obs. Richness = observed richness **C.** PCoA ordination of bacterioplankton samples for high-MP versus low-MP ponds. **D**. PCoA ordination of *Daphnia* host microbiome samples for high-MP versus low-MP ponds. *** - indicate statistical significance of PERMANOVA (**a:** Between *Daphnia* and acterioplankton groups**, C, D:** Between high MP and low MP groups) and Wilcoxon rank-sum test (**B**) at alpha of 0.05.

PERMANOVA showed a significant difference between the composition of bacterioplankton and *Daphnia* microbiome communities assessed with 16S rRNA gene amplicon sequencing (*P* value <.01, *R*^2^ = 0.12, betadisper >0.05) (Fig. 3A). We compared richness (Observed richness, Shannon index) and evenness (Pielou index), finding bacterioplankton more diverse and even (*P* value <.01 for all indices) than the field *Daphnia* microbiomes (Fig. 3B). Assessing each sample type separately, PERMANOVA indicated significant differences in community composition between high and low MP ponds (*P* value <.01, betadisper >0.05) for both bacterioplankton (*R*^2^ = 0.04) and host-associated microbiome (*R*^2^ = 0.09) (Fig. 3C, D). To assess the impact of environmental variables on community composition, db-RDA analysis was applied and revealed that redox (*R*^2^ = 0.05), temperature (*R*^2^ = 0.06), and chlorophyll (*R*^2^ = 0.05) were significant predictors of community composition, with a combined adjusted *R*^2^ of 0.16 (Supplementary Fig. 2). No significant differences in richness and evenness were found between high and low MP samples for either community (Supplementary Fig. 3).

The top 10 most abundant taxa in each sample were visualized using stacked bar charts (Supplementary Fig. 5). Although both bacterioplankton and *Daphnia* microbiome communities shared dominant orders such as *Burkholderiales* and *Flavobacteriales*, notable differences in taxa abundances were observed between pond types. In urban ponds, *Daphnia* hosted higher abundances of chitin-degrading *Chitinophagales* and saprophytic *Mycoplasmatales*, whereas bacterioplankton communities featured more *Pseudomonadales* and *Micrococcales*. Conversely, in natural ponds, *Daphnia* exhibited higher proportions of *Rickettsiales* and *Sphingomonadales*, whereas bacterioplankton displayed greater abundances of *Pirellulales* and *Verrucomicrobiales*.

### Increased abundance of PET catabolic genes and ARGs in microbiomes sampled from MP-rich ponds

**Figure 4:**
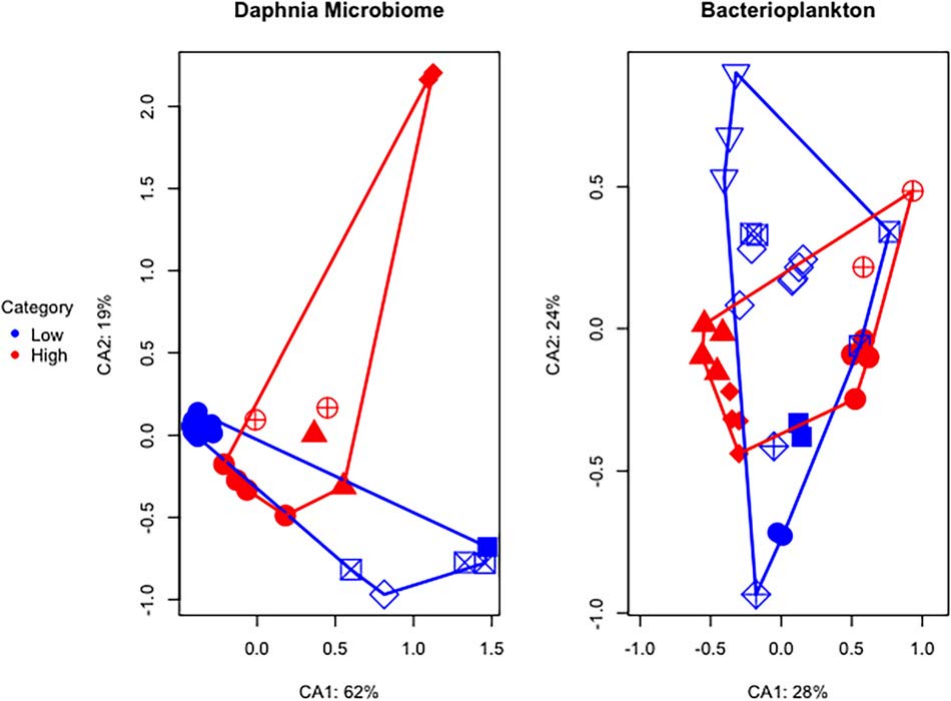
**Differences in gene abundance in bacterioplankton and host microbiome samples.** Constrained ordination (db-RDA) based on Bray-Curtis dissimilarities between metagenome samples, using proportional abundances of gene functions (COGs). The constrained axis CA1/CA2 represent variation explained by the pond MP category (high vs. low MP concentration) and pond location (marked by the shape). *** - indicates significant PERMANOVA test between pond MP categories.

**Figure 5:**
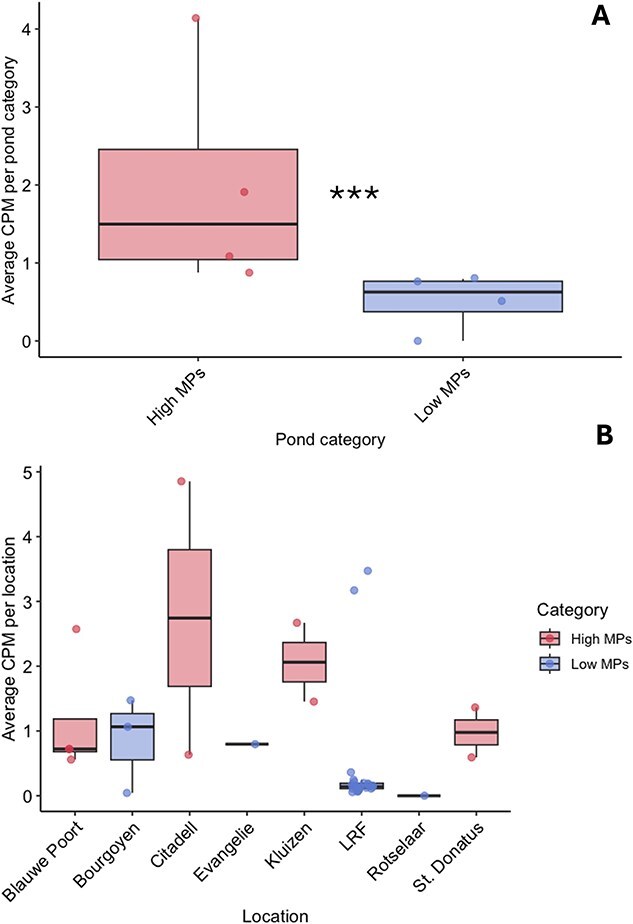
**Difference in abundance of antibiotic resistance genes (ARGs) in the host microbiome between ponds (N = 8) and categories. A.** Box plot showing the distribution of ARGs as counts per million (CPM) per pond category. *** - indicates significant Wilcoxon rank sum test at alpha of 0.05. **B.** Box plot showing the abundance of ARGs as counts per million (CPM) per individual pond.

**Figure 6:**
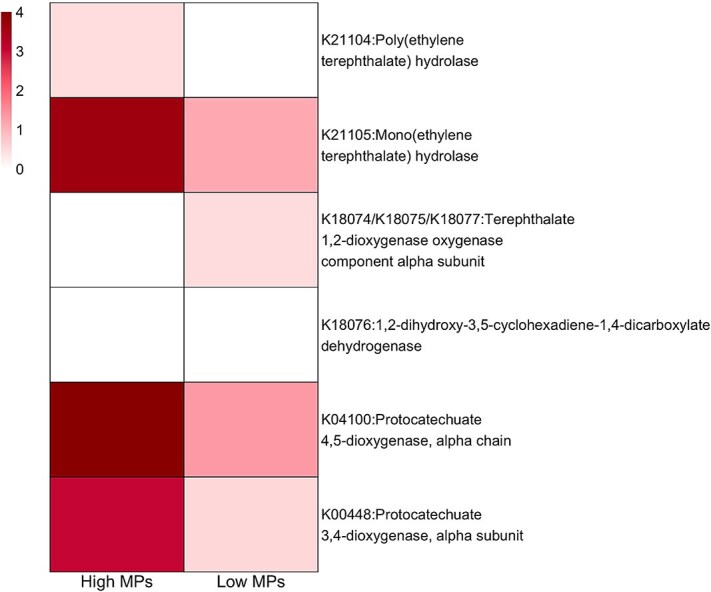
**Heatmap showing abundances of enzymes involved in the PET degradation in host microbiome samples from high versus low MP ponds.** The abundances are expressed as square roots of average CPM (counts per million) reads mapped to KOs involved in the biodegradation of PET. The proteins were mapped to KOs using KEGG mapper version 5.1.

We assessed whether the functional profiles of host microbiome and bacterioplankton samples are significantly different. The PCoA demonstrated a distinct partitioning between microbiome and bacterioplankton samples along the principal components (Fig. 4) and a significant difference between low and high MP catabolic gene composition for microbiome samples (PERMANOVA, *P* value <.05, betadisper >0.05). ARG have been correlated with plastic pollution in previous studies [64], so we quantified their abundance in the host-associated microbiome samples. The analysis of average resistome abundances revealed a statistically significant elevation in ARGs within the microbiome of high MP ponds compared to those from low MP ponds, as depicted in Fig. 5 (Wilcoxon rank sum, *P* value <.05, rank-biserial correlation (*r*) = 0.82). Furthermore, we quantified the genes encoding for the degradation pathway of the PET polymer, including the first two steps: PET hydrolase (PETase) [EC:3.1.1.101] and mono(ethylene terephthalate) hydrolase (MHETase) [EC:3.1.1.102]. The results are presented as a heatmap in Fig. 6 showing mean abundances for each KO enzyme. Gene homologues encoding enzymes known to be involved in bacterial PET metabolism were only found in the high MP pond group, including the genes encoding the initial depolymerase PETase (as well as the MHETase).

### Bacterioplankton inoculum is the most important factor influencing microbiome composition of *Daphnia in presence of MP*

**Figure 7:**
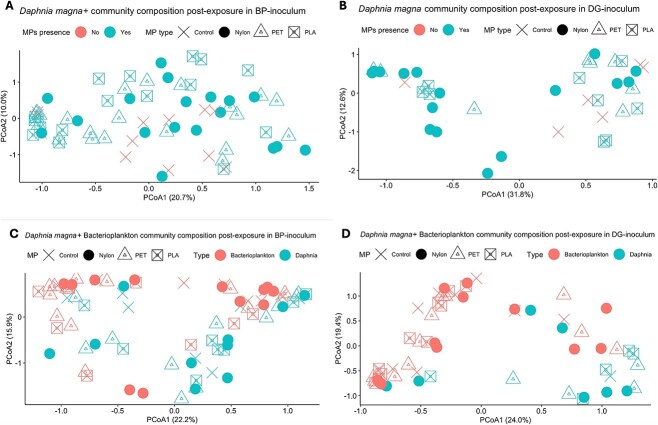
Study of microbiome community composition and diversity in bacterioplankton and *Daphnia* microbiome samples. **A.** PCoA ordination of Bray-Curtis dissimilarities for BP-inoculated *Daphnia magna* samples after the exposure experiment stratified by MP type (C, Nylon, PET, PLA) and MP presence (Yes, No). **B.** PCoA ordination of Bray-Curtis dissimilarities for DG-inoculated *D. magna* samples after the exposure experiment stratified by MP type (C, Nylon, PET, PLA) and MP presence (Yes, No). **C.** PCoA ordination of Bray-Curtis dissimilarities for bacterioplankton samples (red) co-cultured with *D. magna* (blue) from BP-inoculated jars stratified by MP type (shapes) **D.** PCoA ordination of Bray-Curtis dissimilarities for bacterioplankton samples (red) co-cultured with *D. magna* (blue) from BP-inoculated jars stratified by MP type (shapes).

The *in-situ* analysis revealed differences in the microbiome between high and low MP freshwater environments, with the high MP communities showing an association with PET catabolic genes, which may suggest a potential response to MP pollution. To examine effects of MP exposure on *Daphnia* microbiome composition in a controlled environment with previously adapted bacterioplankton inoculum, we conducted a 23-day exposure experiment. Specifically, different *D. magna* genotypes were exposed to high MP concentrations with bacterioplankton inocula from either DG and BP as a low and high MP pond, respectively (Fig. 1E). Treatment groups included three MP fibres (Nylon, PET, PLA), three *Daphnia* genotypes (KNO15.04, F, BH), and two water sources (BP and DG). We first investigated whether MP exposure significantly affected *Daphnia* microbiome composition. No significant difference was found between the microbiomes of non-exposed and MP-exposed *Daphnia* both in BP (PERMANOVA, *P* value >.05, betadisper >0.05, *R*^2^ = 0.02) and DG-inoculated jars (PERMANOVA, *P* value >.05, betadisper >0.05, *R*^2^ = 0.03) (Fig. 7A, B). Next, we compared the microbiome composition of pre-exposure lab *Daphnia* (i.e. clone control) with the exposure controls (post-exposure, no MP added). PERMANOVA revealed a significant difference in composition (*P* value <.05, betadisper >0.05, *R*^2^ = 0.17), suggesting a change in microbiome composition over the course of the experiment, perhaps due to uptake of the surrounding bacterioplankton. However, post-exposure microbiomes remained distinct from their surrounding bacterioplankton (PERMANOVA, *P* value >.05, betadisper >0.05, *P* value <.05, *R*^2^ = 0.07 and *R*^2^ = 0.1 for BP and DG bacterioplankton, respectively) (Fig. 7C, D) and bacterioplankton composition differed significantly between jars with and without *Daphnia* (PERMANOVA, *P* value <.05, betadisper >0.05, *R*^2^ = 0.1 and *R*^2^ = 0.06 for BP and DG bacterioplankton, respectively). This highlights an exchange between *Daphnia* microbiomes and bacterioplankton. Given the lack of effect of MP on microbiome composition, we conducted a multi-factor PERMANOVA to explore other variables that may have influenced the post-exposure microbiomes. The analysis revealed that the inoculum source (pond bacterioplankton) was a significant factor affecting microbiome composition after exposure (PERMANOVA, *P* value <.05, *R*^2^ = 0.07) in both inoculum groups. Moreover, microbiome composition was affected by the age of the individual (juvenile versus adult), but only in the BP inoculated samples. None of the other factors (genotype, MP presence) had a significant effect on microbiome composition post-exposure in both inoculum groups.

### Taxa associated to microplastics

The top 10 most abundant taxa in each sample were visualized using stacked bar charts (Supplementary Fig. 5). The microbial communities of both *Daphnia* and bacterioplankton exhibited significant variations based on pond origin (Blauwe Poort [BP] or DG) and plastic treatments (Nylon, PET, PLA, Control). In *Daphnia*-associated microbiome in DG pond water, Rhodobacterales were highly abundant in the control (71.6%) but decreased with PLA (62.4%), PET (44.5%), and Nylon (37.5%). Conversely, in those in BP pond water, Rhodobacterales were lower in the control (17.6%) but increased with Nylon (40.9%) and PLA (34.1%). Burkholderiales showed higher abundance in BP *Daphnia* controls (26.0%) and decreased in the presence of plastics, whereas in DG pond water, their abundance increased with PLA and Nylon treatments. For bacterioplankton, Rhodobacterales were low in DG control (6.9%) but rose with PET (19.7%) and Nylon treatments (19.1%), and similarly increased in BP pond in the presence of plastics compared to controls. Burkholderiales remained consistently prevalent, especially in BP Nylon (19.4%) and DG PLA (20.9%). Additionally, orders like Flavobacteriales and Planctomycetales were more abundant in specific plastic treatments.

We conducted a network analysis on DG and BP-inoculated *Daphnia* microbiome communities from the exposure experiment to uncover significant taxon-taxon and taxon-MP associations (Supplementary Fig. 4). For the BP community, the genus *Prosthecobacter* was positively associated with PET and the order *Planctomycetales* and the genus *Gemmobacter* positively with Nylon (Supplementary Table 4). We traced the origin of each taxon to either pre-treatment (taxa from the pre-exposure microbiome of laboratory-reared *D. magna*), post-treatment (taxa derived from the bacterioplankton inoculum), or both. *Prosthecobacter* was identified both in the pre-treatment microbiome and BP inocula whereas *Planctomycetales* and *Gemmobacter* were unique to inoculum. The low-MP inoculum (DG) taxa were not associated with any plastic variable.

### MP and bacterioplankton inoculum influence *D. magna* survival

**Figure 8:**
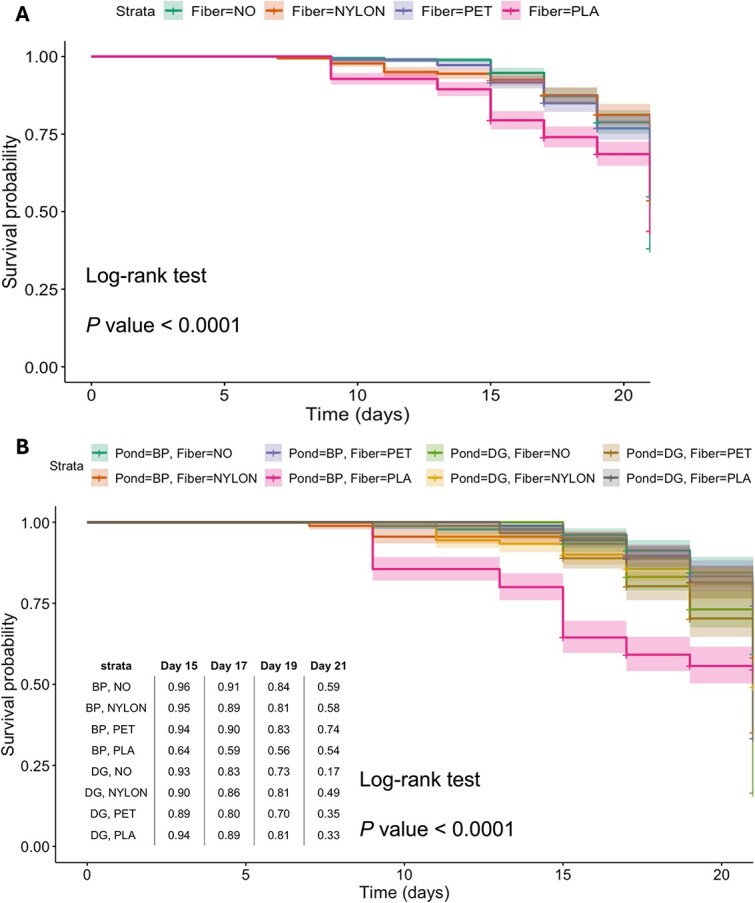
**Survival analysis of *Daphnia magna* over a 23-day exposure period. A.** Kaplan–Meier survival curves for *D. magna* populations stratified by fiber type, where “Fiber = NO” represents the control group. **B.** Kaplan–Meier survival curves illustrating the combined effect of fiber type and pond origin on survival, with log-rank test p-values shown on the plot to indicate significance. The table presents survival probabilities for each experimental group at days 15, 17, 19, and 21, offering additional insight into group-specific survival trends over time.

*Daphnia* life history parameters were tracked throughout the experiment. A significant decline in survival was observed after 20 days, particularly in the PLA-treated groups (Fig. 8A). We examined the combined effects of fiber type and bacterioplankton inoculum (Fiber × Pond) on *Daphnia* survival (Fig. 8B). At the end of the experiment, a sharp decline was observed in all groups, especially in the DG × control group, where survival fell below 25%. The survival of *Daphnia* individuals was higher in groups inoculated with high-MP bacterioplankton (BP × PET, BP × control, BP × PLA, BP × Nylon) compared to low MP-bacterioplankton (DG × Control, DG × PLA, DG × PET, DG × Nylon) (Log-rank test, Chi-squared = 6.3, *P* value <.01). Cox proportional hazards analysis indicated that *Daphnia* inoculated with DG pond bacterioplankton had a 24.8% higher risk of death compared to those inoculated with BP bacterioplankton (Supplementary Fig. 6). PLA fibers were associated with a 47.5% higher risk of death than other fibers and control groups (Supplementary Fig. 8). Analysis of the day of the first brood, an important reproductive parameter, showed no significant difference between *Daphnia* exposed to the DG and BP pond inocula in the presence of plastic, indicating that pond origin did not influence the timing of reproduction in this context.

## Discussion

### Microplastic profiles in urban water bodies: sources, composition, and accumulation

This study highlights the impact of urbanization on MP distribution in aquatic environments, with significantly higher MP concentrations in city pond sediments and water than in natural lakes. Our results are consistent with previous research showing urban water bodies as major MP sinks due to increased human activity [4, 5, 65, 66]. We found a discrepancy in the concentration and composition of MP between sediment and water samples in the urban settings: sediment samples were dominated by polypropylene (PP) and polyethylene (PE), whereas water samples were rich in polyesters, including PET. Plastics denser than water (e.g., nylon, PVC) tend to settle in sediments [67], whereas low-density polymers like PE and PP (0.89–0.97 g/cm^3^) are also commonly found in sediments due to biofouling or the addition of mineral fillers [67–69].These processes will also affect the accumulation rate of MP across the layers. We observed more MP in sediment than in the water column, where biodegradation is slower due to reduced photooxidation [70]. Although PE and PP derive from plastic waste fragmentation, polyesters primarily enter freshwater through wastewater from textile washing [71, 72]. Therefore, despite their higher relative abundance in water, the actual polyester concentration is likely underestimated due to their small size (10–20 μm) being below our 64 μm threshold used in this study [73].

### *Daphnia* microbiome composition reflects a balance between host-specific and environmental bacteria

Previous studies showed that *Daphnia* microbiome composition strongly depends on the diversity of bacteria in the surrounding bacterioplankton [24]. Using 16S rRNA gene amplicon sequencing, we found a significant difference between the composition of the bacterioplankton community and the host microbiome in sampled ponds, irrespective of their contamination status (low MP vs high MP). Moreover, bacterioplankton was on average richer and more diverse than the related host microbiome. This finding is consistent with previous observations in in vitro experiments [74] and was also replicated in the MP exposure experiment showing a significantly altered *D. magna* microbiome composition after 23 days of incubation yet remaining distinct from the surrounding bacterioplankton. The composition of the bacterioplankton also varied depending on whether it was cultured with or without *Daphnia*. This suggests that by incorporating external bacteria via browsing behaviour, the emergent microbiome is a mixed community that comprises both the newly assimilated bacterioplankton and the native microbiome members [24, 26, 74, 75].

### Effects of MP on *Daphnia* microbiome community

Uptake of MP by *Daphnia* was shown in previous in vitro exposure studies [7, 8, 10, 11]. In this study, we compared host microbiome and bacterioplankton communities sampled from ponds burdened with MP pollution with those of lower MP exposure. Significant differences in microbial community composition were found between the two environments although their diversity did not differ significantly. MP has been shown before to alter microbial community composition in sediments [76–78]. However, their effects on host-associated microbiomes, particularly within freshwater environments, remain largely unexplored. Considering the complexity of aquatic and host-associated ecosystems, the presence of MP is unlikely to affect microbial community structure without consideration for other interacting factors including environmental variables. Indeed, urban ponds, categorized in this study as high-MP environments, can differ significantly from non-urban lakes in several aspects including among others nutrient levels, physical disturbances, chemical contamination, and habitat structure [79]. Our findings indicate that *Daphnia* microbiome communities in urban ponds with high MP pollution were correlated with chlorophyll concentration, which is a proxy for the amount of algae, including cyanobacteria. Urban environments often contribute to higher nutrient loads (e.g., from runoff containing fertilizers and pollutants from residential and industrial areas), which can exacerbate algal blooms including *Cyanobacteria* that thrive in nutrient-rich conditions [80]. Exposure to cyanobacteria and their toxins has been shown to disrupt the balance of the gut microbiome in *Daphnia* [26]. *Daphnia* microbiome communities in low-MP ponds were more strongly associated with temperature and oxygen availability indicating that in the absence of additional environmental stress, temperature and oxygen may play a more significant role in shaping microbial communities. Oxygen depletion has also been shown to structure *Daphnia* microbiome communities in experiments [60]. Given our observations in the field study, we aimed to investigate the changes in the *D. magna* microbiome community after exposure to MP while controlling for external variables. A 23-day *in-vitro* exposure of *D. magna* to MP revealed no significant effects of MP fibres (PLA, PET, Nylon) on the community structure. Therefore, based on the evidence presented in this study, we conclude that MP do not significantly impact the overall structure of the *Daphnia* microbiome community. However, a few specific taxa were associated with MP presence in our in vitro study, suggesting that although the broad effects may be minimal, MP could influence certain elements of the microbiome.

### Potential Plastisphere: taxonomic and functional enrichment in the presence of MP

Our network analysis of exposure study samples revealed that a taxon classified as *Prosthecobacter*, a member of both *Daphnia* core microbiome and bacterioplankton inoculum community, was linked to PET. This genus is known to thrive in low-nutrient environments and was observed to be enriched in the presence of other plastic polymers including the deep sea plastisphere [81], PET film in stream water [82] and cellulose acetate polymer surface in a brackish marine environment [83]. However, its association with PET has not been reported before for freshwater environments. In addition, two taxa belonging to the *Gemmobacter* genus and *Plantomycetales* order were linked to Nylon. *Gemmobacter* belongs to the *Rhodobacteraceae* family, which is commonly found on plastic [84, 85] and has been recognized as an initial colonizer during biofilm formation [86]. Moreover, it was observed in biofilms developing on PP films in a Hungarian lake [87]. Not much is known about the *Plantomycetales* order in relation to plastic colonization or degradation except that they were identified together with *Gemmobacter* as members of the freshwater biofilms forming on PP films [87]. Moreover, they were found implicated in biofilm formation on various macroalgae [88, 89]. No taxa associated with PLA were identified.

By applying metagenome shotgun sequencing to field samples, we quantified specific functional genes in *Daphnia* microbiomes that could be associated with MP presence, including ARGs and hydrolytic enzymes involved in polyester biodegradation. Recently, plastispheres have been identified as hotspots for ARGs [12, 64, 90] and hence their higher abundances in the host microbiomes from high-MP ponds was not unexpected. Given that high-MP ponds are close to human activity hubs, they are at greater risk of antibiotic contamination through sewage runoff. Furthermore, the increase of ARGs within host microbiomes in high-MP ponds supports the hypothesis that MP may provide a route for human-derived antibiotics and ARGs to enter and establish within aquatic host microbiomes [35, 91]. Furthermore, we found that the host microbiome community from a high-MP environment was enriched in genes encoding PET catabolism. Polyesters are common in freshwater [77] and were also the most abundant MP type in the urban pond waters in the current study. Therefore, these findings support the hypothesis that biofilm-associated microbes may become part of MP-grazing microbiomes as evidenced by enrichment of plastic/biofilm associated taxa in the microbiome community as well as the increase in genes involved in polyester degradation and antibiotic resistance. However, additional research is required to confirm these interactions and fully understand the mechanisms by which MP-associated biofilms influence host microbiomes in freshwater ecosystems.

### Effects of MP on *D. magna* survival

Finally, we assessed whether bacterioplankton pre-exposed to different levels of MP, *Daphnia* genotype and MP type affected the survival of *D. magna.* The host genotype did not matter, but *Daphnia* exposed to PLA fibres exhibited accelerated mortality and a 48% increased risk of death compared to *Daphnia* exposed to other MP types. PLA particles used in the study measured 50 × 50 μm, as opposed to 50 × 10 μm for PET and Nylon. *Daphnia* ingests larger MP particles at a greater rate than smaller particles [92], suggesting that the PLA particles used in the study might have accumulated faster than the two other MP in the *Daphnia* digestive tract, potentially obstructing it and hindering proper digestion and nutrient absorption, ultimately leading to starvation. Another significant factor is the pond inoculum effect: *Daphnia* cultured with bacterioplankton derived from the MP-rich BP pond had the highest survival rates and those cultured with the MP-poor DG pond were 25% more at risk of death both in MP-exposed and control treatment groups. These results suggest that high-MP environment was more favourable to *Daphnia* survival, potentially due to a more beneficial composition of bacterioplankton. Additionally, microbes capable of metabolizing plastic compounds may enrich the pond's carbon sources, supporting a diverse microbial community that could enhance food availability [93]. However, other pond system characteristics, such as metabolite production, pH shifts, and altered oxygen levels, may also influence survival outcomes. Thus, the role of bacterioplankton in affecting *Daphnia* survival under MP exposure merits further investigation to clarify these mechanisms.

## Conclusions

In conclusion, our study highlights consequences associated with urbanization for aquatic ecosystems such as higher MP and ARG burden. Moreover, our combined in situ and in vitro approach demonstrates the enrichment of plastisphere-associated taxa and plastic-degradation functions in the *Daphnia* microbiome as a result of MP presence. Our research opens avenues to further explore the adaptive mechanisms of aquatic microbiomes and acclimatization of keystone invertebrates in response to MP pollution, emphasizing the complex interactions within ecosystems and the potential long-term impacts of urbanization.

## Supplementary Material

Supplementary_Table_1_wrae234

## Data Availability

*Reads and metadata are available at PRJNA1122644:* Field’s whole genome shotgun reads accessions: SAMN41788498–526. Field’s amplicon 16 rRNA Miseq reads accessions: SAMN41798624–771. MP exposure amplicon 16 rRNA Miseq reads accessions: SAMN41876284–514.

## Scripts

https://github.com/msysbio/PlasticDaphnia-ISMEJ.
